# Supplementary material for: Association between oral microbiome and seven types of cancers in East Asian population: a two-sample Mendelian randomization analysis
Source: Front Mol Biosci. 2023 Nov 21;10:1327893. doi: 10.3389/fmolb.2023.1327893 (PMC10702768; doi:10.3389/fmolb.2023.1327893)
Supplement: Supplementary file 1 [file DataSheet1.ZIP › Supplementary Materials/MR plots of saliva/saliva═╝/Pancreatic cancer/pheno.219_to_pancreatic cancer_leave_one_out.pdf]

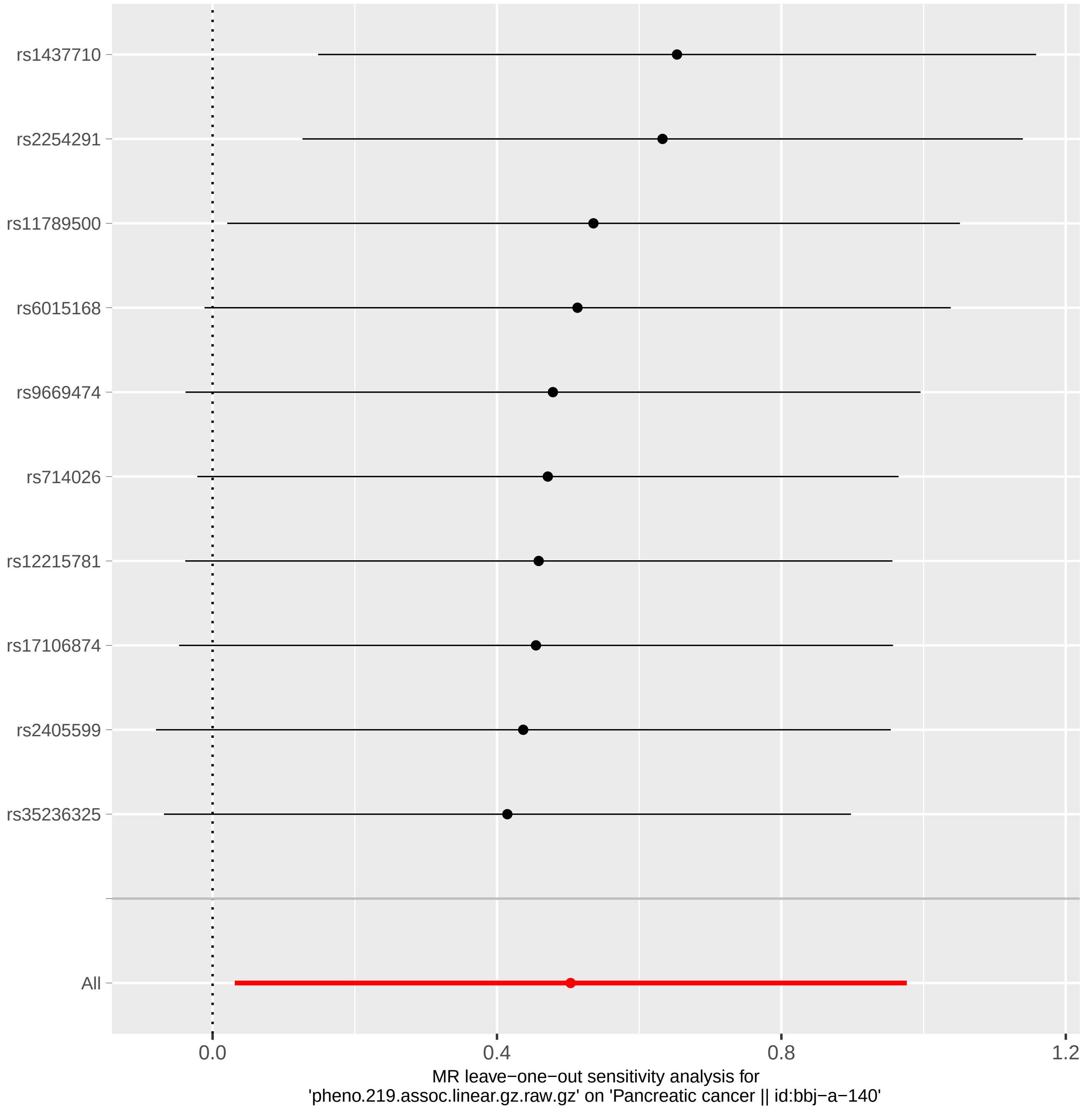
rs1437710
rs2254291
rs11789500
rs6015168
rs9669474
rs714026
rs12215781
rs17106874
rs2405599
rs35236325
All
0.0
0.4
0.8
1.2
MR leave−one−out sensitivity analysis for
'pheno.219.assoc.linear.gz.raw.gz' on 'Pancreatic cancer || id:bbj−a−140'
